# 5-(4-Fluoro­phen­yl)-2,2,6-trimethyl-4*H*-1,3-dioxin-4-one

**DOI:** 10.1107/S1600536809023666

**Published:** 2009-06-27

**Authors:** Julio Zukerman-Schpector, Adriano S. Vieira, Hélio A. Stefani, Edward R. T. Tiekink

**Affiliations:** a Department of Chemistry, Universidade Federal de São Carlos, 13565-905 São Carlos, SP, Brazil; b Departamento de Farmácia, Faculdade de Ciências Farmacêuticas, Universidade de São Paulo, São Paulo-SP, Brazil

## Abstract

The 1,3-dioxine ring in the title compound, C_13_H_13_FO_3_, is in a half-boat conformation with the methyl-bonded C atom 0.612 (2) Å out of the plane defined by the remaining five atoms.

## Related literature

For synthetic and structural background, see: Caracelli *et al.* (2007 ▶); Stefani *et al.* (2007 ▶); Vieira *et al.* (2008 ▶). For conformational analysis, see: Cremer & Pople (1975 ▶); Iulek & Zukerman-Schpector (1997 ▶).
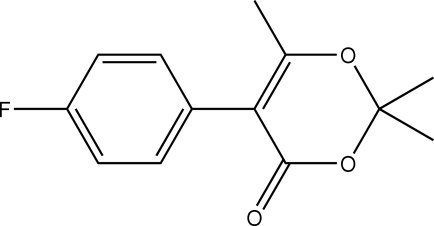


## Experimental

### 

#### Crystal data

C_13_H_13_FO_3_
*M* *_r_* = 236.23
Monoclinic, 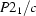

*a* = 11.865 (3) Å
*b* = 7.781 (2) Å
*c* = 12.780 (4) Å
β = 107.369 (5)°
*V* = 1126.1 (5) Å^3^
*Z* = 4
Mo *K*α radiation
μ = 0.11 mm^−1^
*T* = 98 K
0.20 × 0.15 × 0.08 mm

#### Data collection

Rigaku AFC12/SATURN724 diffractometer
Absorption correction: multi-scan (*ABSCOR*; Higashi, 1995 ▶) *T*_min_ = 0.977, *T*_max_ = 1 (expected range = 0.969–0.991)
4071 measured reflections
2058 independent reflections
1895 reflections with *I* > 2σ(*I*)
*R*_int_ = 0.058

#### Refinement

*R*[*F* ^2^ > 2σ(*F* ^2^)] = 0.052
*wR*(*F* ^2^) = 0.150
*S* = 1.15
2058 reflections
157 parameters
H-atom parameters constrained
Δρ_max_ = 0.32 e Å^−3^
Δρ_min_ = −0.25 e Å^−3^

### 

Data collection: *CrystalClear* (Rigaku/MSC 2005 ▶); cell refinement: *CrystalClear*; data reduction: *CrystalClear*; program(s) used to solve structure: *SIR97* (Altomare *et al.*, 1999 ▶); program(s) used to refine structure: *SHELXL97* (Sheldrick, 2008 ▶); molecular graphics: *DIAMOND* (Brandenburg, 2006 ▶); software used to prepare material for publication: *WinGX* (Farrugia, 1999 ▶).

## Supplementary Material

Crystal structure: contains datablocks global, I. DOI: 10.1107/S1600536809023666/ng2601sup1.cif

Structure factors: contains datablocks I. DOI: 10.1107/S1600536809023666/ng2601Isup2.hkl

Additional supplementary materials:  crystallographic information; 3D view; checkCIF report

## Figures and Tables

**Fig. 1.**
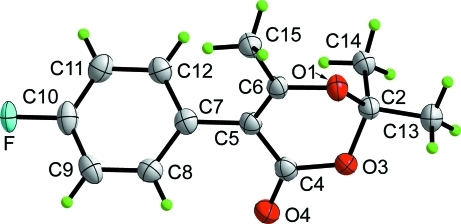
The molecular structure of (I) showing atom labelling scheme and displacement ellipsoids at the 50% probability level (arbitrary spheres for the H atoms).

## supplementary crystallographic information

### Comment

As part of our on-going research interest efforts exploring the chemistry of potassium organotrifluoroborate salts including their potential use as intermediates in organic synthesis (Caracelli *et al.*, 2007; Stefani *et al.*, 2007; Vieira *et al.* 2008), herein the crystal structure of (I) is described. The molecular structure, Fig. 1, shows the six-membered ring to adopt a half-boat conformation with the C2 atom being 0.612 (2) Å out of the plane defined by the remaining five atoms. The ring-puckering parameters being q_2_ = 0.415 (2) Å, q_3_ = 0.189 (1) Å, Q = 0.456 (1) Å, and φ_2_ = 53.3 (2)°. The aryl ring is twisted with respect to the planar portion of the dioxin-4-one ring, as seen in the C4—C5—C7—C8 tosion angle of 55.8 (2)°.

### Experimental

Single crystals of (I) were obtained by slow evaporation from methanol.

### Refinement

The H atoms were positioned with idealized geometry using a riding model with C—H = 0.93–0.96 Å, and with *U*_iso_ set to 1.2 times (1.5 for methyl) *U*_eq_(parent atom).

### Crystal data

**Table:**

C_13_H_13_FO_3_	*F*(000) = 496
*M**_r_* = 236.23	*D*_x_ = 1.393 Mg m^−^^3^
Monoclinic, *P*2_1_/*c*	Mo *K*α radiation, λ = 0.71073 Å
Hall symbol: -P 2ybc	Cell parameters from 2836 reflections
*a* = 11.865 (3) Å	θ = 2.8–40.2°
*b* = 7.781 (2) Å	µ = 0.11 mm^−^^1^
*c* = 12.780 (4) Å	*T* = 98 K
β = 107.369 (5)°	Prism, colourless
*V* = 1126.1 (5) Å^3^	0.20 × 0.15 × 0.08 mm
*Z* = 4	

### Data collection

**Table:**

Rigaku AFC12/SATURN724 diffractometer	2058 independent reflections
Radiation source: fine-focus sealed tube	1895 reflections with *I* > 2σ(*I*)
graphite	*R*_int_ = 0.058
ω scans	θ_max_ = 25.5°, θ_min_ = 3.2°
Absorption correction: multi-scan (*ABSCOR*; Higashi, 1995)	*h* = −14→11
*T*_min_ = 0.977, *T*_max_ = 1	*k* = −6→9
4071 measured reflections	*l* = −10→15

### Refinement

**Table:**

Refinement on *F*^2^	Primary atom site location: structure-invariant direct methods
Least-squares matrix: full	Secondary atom site location: difference Fourier map
*R*[*F*^2^ > 2σ(*F*^2^)] = 0.052	Hydrogen site location: inferred from neighbouring sites
*wR*(*F*^2^) = 0.150	H-atom parameters constrained
*S* = 1.15	*w* = 1/[σ^2^(*F*_o_^2^) + (0.0872*P*)^2^ + 0.1727*P*] where *P* = (*F*_o_^2^ + 2*F*_c_^2^)/3
2058 reflections	(Δ/σ)_max_ < 0.001
157 parameters	Δρ_max_ = 0.32 e Å^−^^3^
0 restraints	Δρ_min_ = −0.25 e Å^−^^3^

### Special details

**Table:**

Geometry. All s.u.'s (except the s.u. in the dihedral angle between two l.s. planes) are estimated using the full covariance matrix. The cell s.u.'s are taken into account individually in the estimation of s.u.'s in distances, angles and torsion angles; correlations between s.u.'s in cell parameters are only used when they are defined by crystal symmetry. An approximate (isotropic) treatment of cell s.u.'s is used for estimating s.u.'s involving l.s. planes.
Refinement. Refinement of *F*^2^ against ALL reflections. The weighted *R*-factor *wR* and goodness of fit *S* are based on *F*^2^, conventional *R*-factors *R* are based on *F*, with *F* set to zero for negative *F*^2^. The threshold expression of *F*^2^ > σ(*F*^2^) is used only for calculating *R*-factors(gt) *etc*. and is not relevant to the choice of reflections for refinement. *R*-factors based on *F*^2^ are statistically about twice as large as those based on *F*, and *R*- factors based on ALL data will be even larger.

### Fractional atomic coordinates and isotropic or equivalent isotropic displacement parameters (Å^2^)

**Table:**

	*x*	*y*	*z*	*U*_iso_*/*U*_eq_	
C2	0.63242 (13)	−0.0457 (2)	0.70282 (12)	0.0194 (4)	
C4	0.70622 (13)	−0.1569 (2)	0.56052 (13)	0.0193 (4)	
C5	0.71736 (13)	0.0244 (2)	0.53203 (12)	0.0186 (4)	
C6	0.65075 (13)	0.1421 (2)	0.56386 (12)	0.0184 (4)	
C7	0.79551 (13)	0.0670 (2)	0.46347 (12)	0.0192 (4)	
C8	0.77995 (14)	−0.0091 (2)	0.36116 (13)	0.0208 (4)	
H8	0.7177	−0.0854	0.3339	0.025*	
C9	0.85535 (14)	0.0266 (2)	0.29960 (13)	0.0237 (4)	
H9	0.8447	−0.0247	0.2316	0.028*	
C10	0.94636 (15)	0.1401 (2)	0.34182 (14)	0.0244 (4)	
C11	0.96583 (14)	0.2194 (2)	0.44186 (14)	0.0240 (4)	
H11	1.0281	0.2960	0.4679	0.029*	
C12	0.88968 (14)	0.1817 (2)	0.50293 (13)	0.0216 (4)	
H12	0.9014	0.2333	0.5710	0.026*	
C13	0.53730 (14)	−0.0969 (2)	0.75301 (13)	0.0229 (4)	
H13A	0.5194	−0.0014	0.7929	0.034*	
H13B	0.5644	−0.1918	0.8021	0.034*	
H13C	0.4676	−0.1301	0.6960	0.034*	
C14	0.74870 (14)	−0.0007 (2)	0.78687 (13)	0.0224 (4)	
H14A	0.8081	0.0157	0.7507	0.034*	
H14B	0.7720	−0.0924	0.8391	0.034*	
H14C	0.7397	0.1033	0.8241	0.034*	
C15	0.63020 (15)	0.3252 (2)	0.52936 (13)	0.0220 (4)	
H15A	0.6699	0.3502	0.4758	0.033*	
H15B	0.6605	0.3984	0.5920	0.033*	
H15C	0.5470	0.3450	0.4982	0.033*	
O1	0.58727 (9)	0.09857 (15)	0.63304 (9)	0.0209 (3)	
O3	0.64762 (9)	−0.18821 (14)	0.63603 (9)	0.0198 (3)	
O4	0.73951 (10)	−0.27917 (15)	0.51946 (9)	0.0251 (3)	
F	1.02109 (9)	0.17542 (15)	0.28148 (9)	0.0337 (3)	

### Atomic displacement parameters (Å^2^)

**Table:**

	*U*^11^	*U*^22^	*U*^33^	*U*^12^	*U*^13^	*U*^23^
C2	0.0239 (8)	0.0180 (8)	0.0181 (8)	0.0005 (6)	0.0090 (6)	0.0009 (6)
C4	0.0173 (8)	0.0235 (8)	0.0169 (7)	−0.0010 (6)	0.0046 (6)	−0.0016 (6)
C5	0.0192 (7)	0.0209 (8)	0.0152 (8)	−0.0004 (6)	0.0042 (6)	0.0001 (6)
C6	0.0175 (8)	0.0226 (8)	0.0153 (8)	−0.0019 (6)	0.0049 (6)	−0.0002 (6)
C7	0.0194 (8)	0.0195 (8)	0.0182 (8)	0.0042 (6)	0.0048 (6)	0.0034 (6)
C8	0.0215 (8)	0.0197 (8)	0.0208 (8)	0.0021 (6)	0.0056 (6)	0.0005 (6)
C9	0.0266 (8)	0.0280 (9)	0.0175 (8)	0.0073 (7)	0.0083 (6)	0.0031 (7)
C10	0.0211 (8)	0.0305 (9)	0.0246 (9)	0.0076 (6)	0.0115 (7)	0.0092 (7)
C11	0.0190 (8)	0.0264 (8)	0.0257 (9)	−0.0003 (6)	0.0051 (6)	0.0043 (7)
C12	0.0211 (8)	0.0242 (8)	0.0188 (8)	0.0021 (6)	0.0050 (6)	0.0019 (6)
C13	0.0231 (8)	0.0257 (9)	0.0222 (8)	−0.0009 (6)	0.0104 (7)	0.0022 (7)
C14	0.0253 (8)	0.0252 (9)	0.0181 (8)	−0.0027 (6)	0.0085 (6)	−0.0005 (6)
C15	0.0257 (8)	0.0210 (8)	0.0212 (8)	0.0017 (6)	0.0098 (6)	0.0010 (6)
O1	0.0231 (6)	0.0218 (6)	0.0205 (6)	0.0022 (5)	0.0106 (5)	0.0030 (5)
O3	0.0237 (6)	0.0181 (6)	0.0192 (6)	−0.0014 (4)	0.0090 (5)	−0.0014 (5)
O4	0.0313 (7)	0.0208 (6)	0.0265 (7)	0.0005 (5)	0.0140 (5)	−0.0027 (5)
F	0.0276 (6)	0.0474 (7)	0.0327 (6)	0.0013 (5)	0.0191 (5)	0.0079 (5)

### Geometric parameters (Å, °)

**Table:**

C2—O1	1.4345 (19)	C9—H9	0.9300
C2—O3	1.4429 (19)	C10—F	1.3655 (19)
C2—C13	1.509 (2)	C10—C11	1.375 (3)
C2—C14	1.515 (2)	C11—C12	1.390 (2)
C4—O4	1.2081 (19)	C11—H11	0.9300
C4—O3	1.3691 (18)	C12—H12	0.9300
C4—C5	1.473 (2)	C13—H13A	0.9600
C5—C6	1.349 (2)	C13—H13B	0.9600
C5—C7	1.491 (2)	C13—H13C	0.9600
C6—O1	1.3643 (18)	C14—H14A	0.9600
C6—C15	1.490 (2)	C14—H14B	0.9600
C7—C8	1.397 (2)	C14—H14C	0.9600
C7—C12	1.401 (2)	C15—H15A	0.9600
C8—C9	1.385 (2)	C15—H15B	0.9600
C8—H8	0.9300	C15—H15C	0.9600
C9—C10	1.374 (3)		
			
O1—C2—O3	108.85 (12)	C10—C11—C12	118.05 (16)
O1—C2—C13	106.44 (12)	C10—C11—H11	121.0
O3—C2—C13	106.87 (12)	C12—C11—H11	121.0
O1—C2—C14	110.59 (13)	C11—C12—C7	120.97 (15)
O3—C2—C14	110.43 (12)	C11—C12—H12	119.5
C13—C2—C14	113.46 (13)	C7—C12—H12	119.5
O4—C4—O3	117.84 (14)	C2—C13—H13A	109.5
O4—C4—C5	125.57 (15)	C2—C13—H13B	109.5
O3—C4—C5	116.51 (14)	H13A—C13—H13B	109.5
C6—C5—C4	118.14 (14)	C2—C13—H13C	109.5
C6—C5—C7	123.34 (15)	H13A—C13—H13C	109.5
C4—C5—C7	118.39 (14)	H13B—C13—H13C	109.5
C5—C6—O1	120.89 (14)	C2—C14—H14A	109.5
C5—C6—C15	128.21 (15)	C2—C14—H14B	109.5
O1—C6—C15	110.85 (13)	H14A—C14—H14B	109.5
C8—C7—C12	118.39 (14)	C2—C14—H14C	109.5
C8—C7—C5	121.63 (14)	H14A—C14—H14C	109.5
C12—C7—C5	119.94 (14)	H14B—C14—H14C	109.5
C9—C8—C7	121.29 (15)	C6—C15—H15A	109.5
C9—C8—H8	119.4	C6—C15—H15B	109.5
C7—C8—H8	119.4	H15A—C15—H15B	109.5
C10—C9—C8	118.12 (15)	C6—C15—H15C	109.5
C10—C9—H9	120.9	H15A—C15—H15C	109.5
C8—C9—H9	120.9	H15B—C15—H15C	109.5
F—C10—C9	118.29 (16)	C6—O1—C2	114.91 (12)
F—C10—C11	118.53 (15)	C4—O3—C2	117.38 (12)
C9—C10—C11	123.18 (15)		
			
O4—C4—C5—C6	163.49 (15)	C8—C9—C10—C11	0.0 (3)
O3—C4—C5—C6	−13.0 (2)	F—C10—C11—C12	−179.58 (14)
O4—C4—C5—C7	−12.6 (2)	C9—C10—C11—C12	0.3 (3)
O3—C4—C5—C7	170.94 (12)	C10—C11—C12—C7	−0.3 (2)
C4—C5—C6—O1	8.8 (2)	C8—C7—C12—C11	0.2 (2)
C7—C5—C6—O1	−175.29 (13)	C5—C7—C12—C11	178.08 (14)
C4—C5—C6—C15	−168.39 (15)	C5—C6—O1—C2	25.3 (2)
C7—C5—C6—C15	7.5 (3)	C15—C6—O1—C2	−157.02 (13)
C6—C5—C7—C8	−120.06 (18)	O3—C2—O1—C6	−52.77 (16)
C4—C5—C7—C8	55.8 (2)	C13—C2—O1—C6	−167.62 (12)
C6—C5—C7—C12	62.1 (2)	C14—C2—O1—C6	68.72 (16)
C4—C5—C7—C12	−122.05 (16)	O4—C4—O3—C2	165.97 (14)
C12—C7—C8—C9	0.1 (2)	C5—C4—O3—C2	−17.28 (18)
C5—C7—C8—C9	−177.78 (14)	O1—C2—O3—C4	48.96 (16)
C7—C8—C9—C10	−0.2 (2)	C13—C2—O3—C4	163.53 (12)
C8—C9—C10—F	179.84 (14)	C14—C2—O3—C4	−72.62 (16)
